# Post-orchidectomy retroperitoneal seminoma: A case report

**DOI:** 10.3892/ol.2013.1152

**Published:** 2013-01-23

**Authors:** PIERO CAGLIÀ, ANGELO TRACIA, ROSITA A. CONDORELLI, ALDO E. CALOGERO, ENZO VICARI, MASSIMILIANO VEROUX, CORRADO AMODEO, YLENIA DUCA, LUCIANO TRACIA, ANTONINO F. ARCORIA, CHIARA NICOLETTI, LAURA MONGIOÌ, SANDRO LA VIGNERA

**Affiliations:** 1 Departments of Surgical Sciences, Organ Transplantation and Advanced Technologies;; 2 Medical and Pediatric Sciences, Catania University, Catania, Italy; 3 Plastic Surgery, Catania University, Catania, Italy

**Keywords:** retroperitoneal seminoma, orchidectomy, late relapse

## Abstract

Between 2 and 5% of malignant germ cell tumors in males arise at extragonadal sites. The origin of extragonadal retroperitoneal germ cell tumors remains controversial. Whether these develop primarily in the retroperitoneum or are metastases of a primary testicular tumor has long been debated. We report a 38-year-old male who presented with abdominal pain and was diagnosed with retroperitoneal seminoma. The patient gave a history of having undergone a right orchidectomy for an undescended testis via the inguinal route 10 years previously with a reported histology of benign inflammatory mass.

## Introduction

The incidence of testicular cancer has doubled in the past 30 years and continues to increase (1–3). Testicular tumors account for 1–2% of all malignancies but are among the most common types of cancer in young males (4–7). It is well known that cryptorchidism is a risk factor in the development of germ cell tumors (8,9) and approximately 10% of all testicular tumors arise in an undescended testicle (10). Seminoma is the most common histology (4,11). In contrast to patients with scrotal seminoma, clinical studies report a high proportion of advanced stage disease in patients with abdominal or inguinal seminoma. Data in the literature on cryptorchid seminomas are rare and the numbers of patients in published series are small. Several studies have suggested that early orchidopexy reduces the risk of seminoma de velopment (11–13). Seminoma arising in an undescended inguinal testicle is extremely rare in developed countries due to the standard practice of orchidopexy in the early years of life and orchiectomy in post-adolescent patients with undescended testicles to prevent cancer and infertility. Despite these preventative measures, cases of intra-abdominal testicular tumors occasionally occur in adults. The origin of primary extragonadal gem cell tumors is a matter of debate. Although they may arise virtually anywhere, typically they are found in the midline where they present as retroperitoneal, mediastinal or pineal masses. It remains uncertain, however, whether such tumors develop primarily at extragonadal sites or represent metastases of a primary testicular tumor (14–19).

## Case report

**Figure 1:**
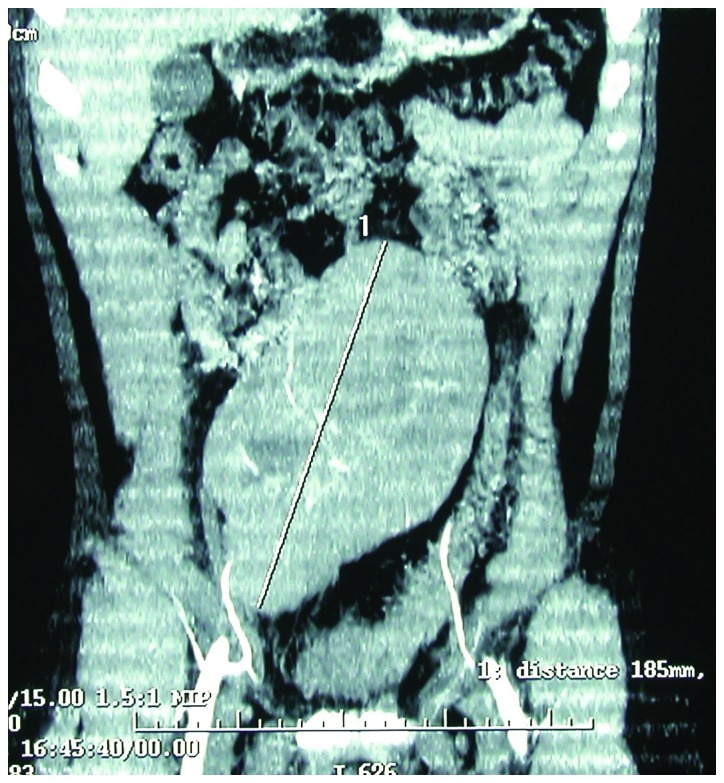
Abdominal computerized tomography shows a solid mass located in the right iliac fossa.

**Figure 2:**
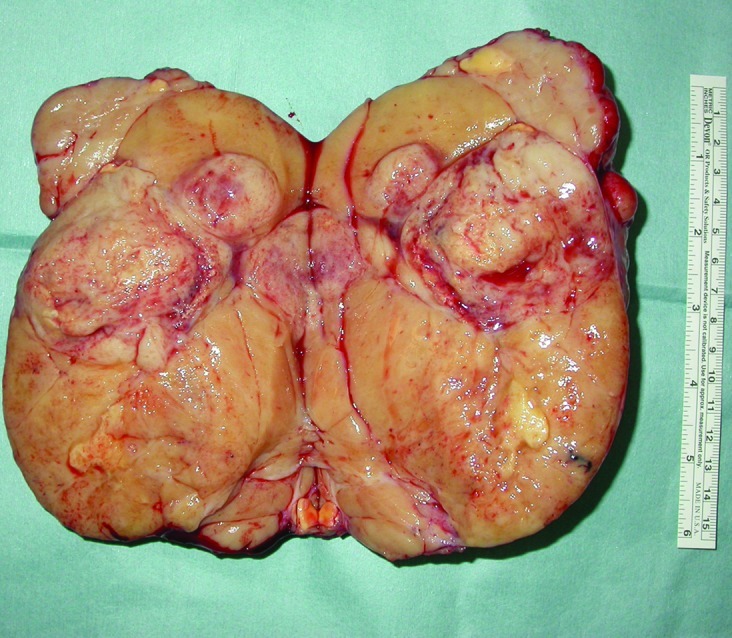
Cut surface of the tumor shows greyish white tissue, cystic areas and a central fibrous core.

We report a 38-year-old male who was referred for abdominal pain to our surgical department. The study was approved by the Institutional Review Board of Catania University, Catania, Italy. Written informed consent was obtained from the patient. Physical examination revealed a well-nourished young male with a palpable mass on the right iliac fossa and the absence of the right testicle. The left spermatic cord and testis were palpable and normal on clinical examination. An incisional scar on the right groin was observed. The patient gave a history of having undergone a right orchidectomy for an undescended testis via the inguinal route 10 years previously. The patient, however, claimed that the histology report described a ‘benign inflammatory mass’. He had remained well until 3 months prior to presentation when he noticed the insidious onset of abdominal pain that progressively increased. No lower urinary or constitutional symptoms were reported and there was no history of trauma to the area. There was no palpable inguinal, axillary or supraclavicular lymphadenopathy. Laboratory studies upon admission revealed a normal blood cell count and chemistry profile. Serum α-fetoprotein (AFP) and β-human chorionic gonadotropin (HCG) were normal. The chest X-ray was also normal. Scrotal ultrasonography revealed the absence of the right testis and that the left testicle, kidney, spleen and liver were in normal condition. Abdominal ultrasonography revealed a heterogeneous mass suggestive of an encapsulated abscess. It was complemented by abdominal computerized tomography that revealed a solid mass located in the right iliac fossa (Fig. 1). There was no presence of retroperitoneal or mesenteric lymph node involvement. The patient underwent a laparotomy that confirmed the diagnosis of a retroperitoneal tumor. At surgery the tumor was almost encapsulated and the resection was performed without complications. The tumor was removed totally. The excised mass measured 18×10×8 cm. Its cut surface showed greyish white tissue, cystic areas and a central fibrous core (Fig. 2). Frozen-section diagnosis indicated that it may be a germ cell tumor. A permanent section revealed that the tumor consisted of round and polygonal cells. The cells had a clear or granular cytoplasm, with a large, centrally located nucleus and coarse-clumped chromatin. There were no non-seminomatous components in the tumor. The histopathology report confirmed the mass to be a classical pure seminoma. Post-operative recovery was good. The patient was referred to receive adjuvant chemotherapy and external beam radiotherapy. The patient was alive and well, without evidence of disease, 2 years and 6 months after the surgery.

## Discussion

Testicular cancer represents 1–2% of all male malignant tumors and is the most frequent solid neoplasia in males aged 20–35 years (5–7). Gonadal dysgenesis, testicular atrophy and trauma have been proposed as etiological factors in the development of testicular cancer but cryptorchidism and a history of tumor in the contralateral testicle are the most significant (11). In cryptorchidic testicles the incidence of testicular cancer is greater than that in the general population (10).

Cryptorchidism is a frequent pathology that affects 2–5% of children. In only in 20% of the cases of cryptorchidism are the testicles non-palpable at clinical examination, in the remaining cases they are in the abdomen or the inguinal canal (9).

Abdominal testicles present a higher rate of malignancy and develop cancer in 30% of cases. The high temperatures in the abdomen and inguinal canal and maternal risk factors appear to be responsible for the malignant degeneration (20). The testicle should be located in patients with non-palpable testicles and orchidectomy is recommended in post-adolescent patients. An exploratory laparoscopy that is, in most cases, simultaneously diagnostic and therapeutic may be performed (9,21).

The histopathology of undescended testicle tumors in the adult depends on location; it is pure seminoma in more than 90% of the cases when it is intra-abdominal (8,21–23). Moreover the prognosis depends on the initial stage and tumor histology (24–26). Orchidopexy does not eliminate the cancer risk but allows an early diagnosis as a result of the testicle being accessible to clinical examination (11). Numerous studies have underlined the association of cancer and cryptorchidism but there is no long series of studies concerning the intra-abdominal localization of testicular tumors in the literature, with the exception of sporadic case reports. Despite the use of chemotherapy, approximately 4% of patients with extragonadal germ cell tumors develop a metachronous testicular cancer (27,28). However, there is disagreement over whether the extragonadal germ cell tumor is a primary disease or metastatic from the burned-out primary testicular lesion (29,30).

No cases of abdominal seminoma following orchidectomy have been reported in the surgical literature. As to whether the resected tumor in the present report was primary or a local recurrence, it would appear that the latter is the case as there are no known structures that may give rise to such a tumor following inguinal orchidectomy. It is possible that a microscopic deposit survived and gave rise to the mass in question, thus calling into question the accuracy of the first pathology report. Post-orchidectomy evaluation of the surgical pathology specimen and the definitive removal of the residual testicle tissue is established by an awareness of the histological spectrum exhibited by testicular remnants (31). Although the exact extragonadal germ cell tumor histogenesis is not known, several theories relating to the possibility that all germ cell tumors originated from extragonadal, potentially biphasic germ cells have been proposed. It has been suggested that germ cells are present in apparently ectopic sites in all healthy individuals, having been distributed widely during normal embryogenesis, conveying genetic information or providing regulatory functions at somatic sites (16,32–34).

As a conclusion, and despite the elevated risk of testicular cancer in patients with intra-abdominal testicles, we considered retroperitoneal seminoma to be a disease of low incidence. This is due to the standard practice of orchidopexy in pre-adolescent patients and orchiectomy in post-adolescents with cryptorchidism. Nevertheless, tumors of intra-abdominal testicles occasionally appear that could be avoided by the adoption of adequate prevention strategies and a careful follow-up. If a patient presents with an abdominal mass following orchidectomy, the differential diagnosis of seminoma should be considered even if the prior histopathology report stated it to be benign. Extirpation of such tumors may be carried out safely by laparotomy. This will obviate the possible rupture and local seeding of tumor cells. We also wish to highlight the fact that a greater number of sections should be taken when preparing histopathological slides of post-orchidectomy testicles to reduce the risk of missing areas of the tumor.
